# A Rare Case of Composite Dural Extranodal Marginal Zone Lymphoma and Chronic Lymphocytic Leukemia/Small Lymphocytic Lymphoma

**DOI:** 10.3389/fneur.2018.00267

**Published:** 2018-04-24

**Authors:** Mark Bustoros, Benjamin Liechty, David Zagzag, Cynthia Liu, Timothy Shepherd, Deborah Gruber, Bruce Raphael, Dimitris G. Placantonakis

**Affiliations:** ^1^Department of Neurosurgery, NYU School of Medicine, New York, NY, United States; ^2^Department of Pathology, NYU School of Medicine, New York, NY, United States; ^3^Perlmutter Cancer Center, NYU Langone Medical Center, New York, NY, United States; ^4^Brain Tumor Center, NYU Langone Medical Center, New York, NY, United States; ^5^Department of Radiology, NYU School of Medicine, New York, NY, United States; ^6^Department of Neurology, NYU School of Medicine, New York, NY, United States; ^7^Department of Medicine, NYU School of Medicine, New York, NY, United States; ^8^Kimmel Center for Stem Cell Biology, NYU School of Medicine, New York, NY, United States; ^9^Neuroscience Institute, NYU School of Medicine, New York, NY, United States

**Keywords:** extranodal marginal zone lymphoma, central nervous system lymphoma, dura, chronic lymphocytic leukemia/small lymphocytic lymphoma, composite lymphoma

## Abstract

**Background:**

Primary extranodal marginal zone lymphoma (MZL) of the dura is a rare neoplastic entity in the central nervous system (CNS).

**Methods:**

We used literature searches to identify previously reported cases of primary dural MZL. We also reviewed clinical, pathologic, and radiographic data of an adult patient with concurrent dural MZL and chronic lymphocytic leukemia (CLL)/small lymphocytic lymphoma (SLL).

**Results:**

We identified 104 cases of dural MZL in the literature. None of them presented concurrently with another type of non-Hodgkin lymphoma. This is the first report of composite lymphoma consisting of dural MZL and CLL/SLL in the bone marrow and lymph nodes.

**Conclusion:**

Primary dural MZL is a rare, indolent low-grade CNS lymphoma, with a relatively good prognosis. Its treatment is multidisciplinary and often requires surgical intervention due to brain compression, along with low to moderate doses of radiotherapy and/or systemic chemotherapy.

## Introduction

Primary central nervous system (CNS) lymphomas account for 3–4% of all CNS malignancies (1–3). Marginal zone lymphoma (MZL) is a low-grade non-Hodgkin’s lymphoma (NHL), which, according to the WHO classification, is subdivided into three types: extranodal (also known as mucosa-associated lymphoid tissue MZL), nodal, and splenic (4). Extranodal MZL is the most common variant. According to recent statistics, it constitutes around 8% of NHL (5), an increase of 3% compared with prior estimates (4). Extranodal MZL of the gastrointestinal tract was first described by Isaacson and Wright as an indolent low-grade lymphoma (6). Although the stomach is the most common site, extranodal MZL also occurs in the mucosa of other organs, including lung, salivary glands, bladder and lacrimal glands, as well as tissues without mucosa, such as thyroid gland, breast, skin, and less frequently the CNS (7–9).

A small number of cases have been reported on dural MZL, all of which were confined to the meninges with no systemic spread at the time of presentation. In this study, we review the literature on dural MZL and present clinical, pathologic, and radiographic data of an adult male with newly diagnosed primary dural MZL and simultaneous chronic lymphocytic leukemia (CLL)/small lymphocytic lymphoma (SLL). The patient subsequently developed lymph node infiltration by both MZL and SLL lymphomas. This case represents the first report of composite lymphoma consisting of dural MZL and CLL/SLL. Composite lymphoma is a rare phenomenon that is defined as the presence of two distinct types of lymphomas in the same patient (10).

## Materials and Methods

### Literature Search

Using PubMed searches, we identified 104 cases of dural MZL worldwide. The case we encountered at our institution is the 105th and the first to present concurrently with CLL/SLL in the bone marrow and peripheral blood investigations.

### Flow Cytometry and Immunohistochemistry (IHC)

Specimens including the patient tumor, peripheral blood, and bone marrow biopsy were submitted for flow cytometry and IHC.

Immunophenotypic markers used in flow cytometry were as follows:

B cells: CD10, CD19, CD20, CD23, kappa, lambda;
T cells: CD2, CD3, CD4, CD5, CD7, CD8;
Myeloid: CD11c, CD13, CD14, CD15, CD33, CD117;
Others: CD103, CD25, CD34, CD36, CD38, CD45, CD56, CD64 FITC, HLA-DR.

The following immunohistochemical stains were performed on the brain specimen:

CD3, CD20, CD21, CD10, CD79a, CD138, Bcl2, Bcl6, MUM-1, Ki-67, *in situ* hybridization studies for kappa, lambda light chains, and EBER;
Immunohistochemistry on the bone marrow biopsy included staining for CD3, CD20, CD5, CD23 and CyclinD1.

### Immunoglobulin (Ig) Gene Rearrangement Analysis

Heavy chain Ig gene rearrangement studies were performed by polymerase chain reaction (PCR) on genomic DNA extracted from tissue. Specific oligonucleotide primers recognizing framework 2 and 3 and the joining (Jh) regions of the human heavy chain were used. PCR products were then electrophoresed on agarose gels. Kappa light chain Ig gene rearrangement analysis was performed using the Invivoscribe IGK Gene Clonality Assay.

## Results

### Case Presentation

**Figure 1:**
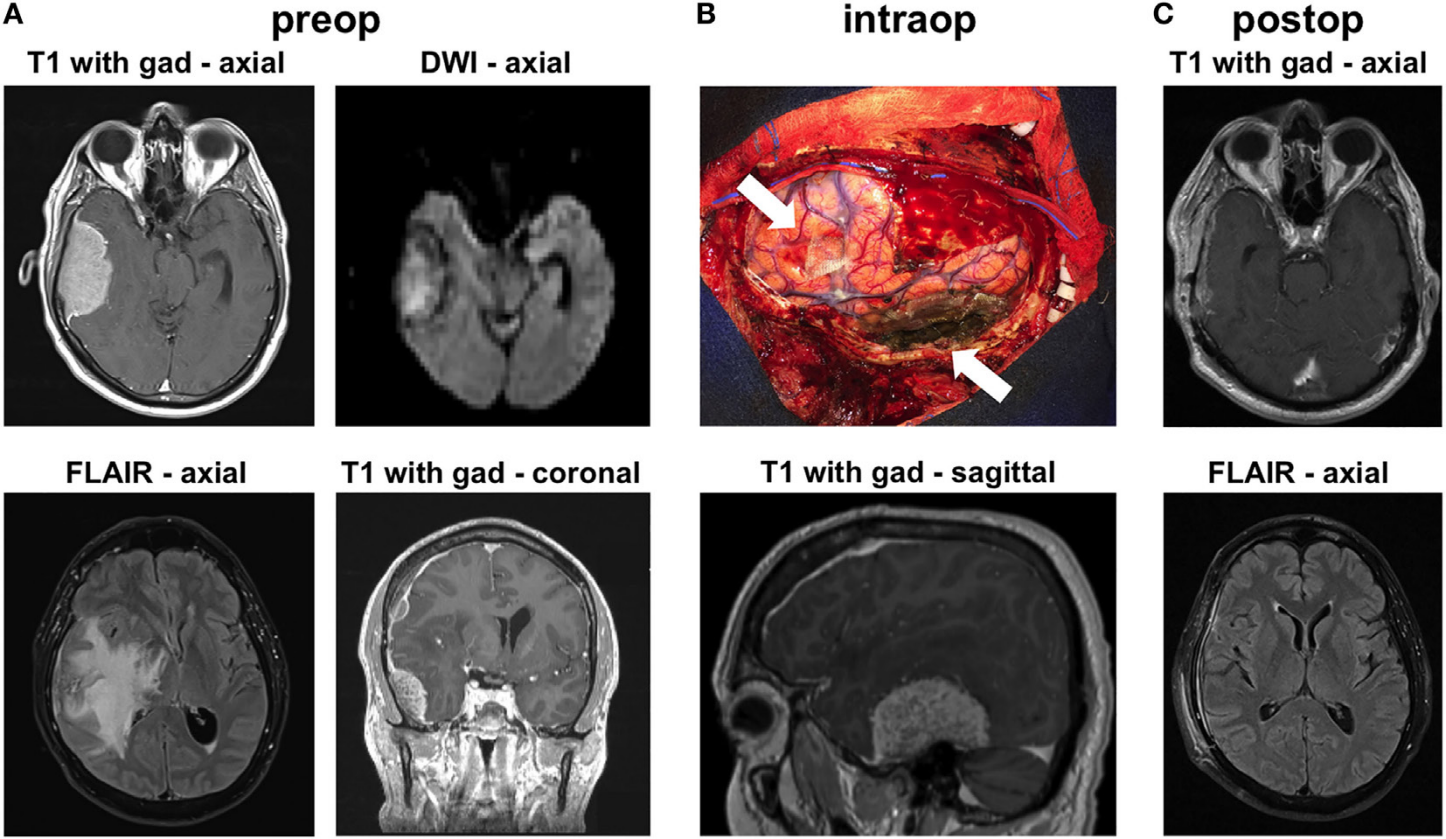
Radiographic findings. **(A)** Preoperative MRI shows contrast-enhancing extra-axial masses in the right temporal and frontal regions. The temporal mass shows diffusion restriction on diffusion-weighted imaging (DWI) imaging. There was substantial vasogenic edema within a large territory of the right hemisphere on FLAIR images. **(B)** Intraoperative image shows the areas of the two masses (arrows), after their resection. The image is shown in conjunction with a preoperative sagittal MRI image that highlights the location of the large temporal mass. **(C)** MRI 6 weeks after surgery shows no recurrence and resolution of the vasogenic edema on FLAIR imaging.

A left-handed 59-year-old male presented at our institution with headaches, imbalance, and a left pronator drift on examination. Brain MRI showed a 6-cm enhancing right temporal mass causing severe brain compression, massive amounts of vasogenic edema and significant right-to-left midline shift (Figure 1A). The mass appeared extra-axial and dural based. The mass also showed diffusion restriction on diffusion-weighted imaging (Figure 1A). He also had a smaller right frontal convexity dural-based lesion with mild local mass effect on the underlying brain parenchyma (Figure 1A). The MRI indicated diffuse pachymeningeal enhancement, especially over the right hemisphere. Given the size of the lesions, as well as the brain compression and the need for diagnosis, we offered the patient a right frontotemporal craniotomy for resection of the two extra-axial lesions. The differential diagnosis preoperatively included multiple meningiomas, metastatic disease, IgG4 disease, and lymphoma.

Intraoperatively, we found that both masses were densely adherent to the dura. We performed gross total resection of both lesions (Figure 1B). The convexity dura attached to the lesions was excised. The dura along the floor of the right middle fossa, where the large temporal mass was attached, was thoroughly coagulated. We used meticulous microdissection to develop the margins of the temporal tumor from the adjacent temporal lobe. Frozen sections from both lesions came back suggestive of atypical lymphoid tissue generating this tumor. Postoperative MRI confirmed gross total resection of both lesions. The patient was started on steroids postoperatively and was discharged to home 3 days after surgery, with complete resolution of his preoperative symptoms. MRI done 6 weeks later showed no recurrence and dramatic improvement in the surrounding vasogenic edema (Figure 1C).

**Figure 2:**
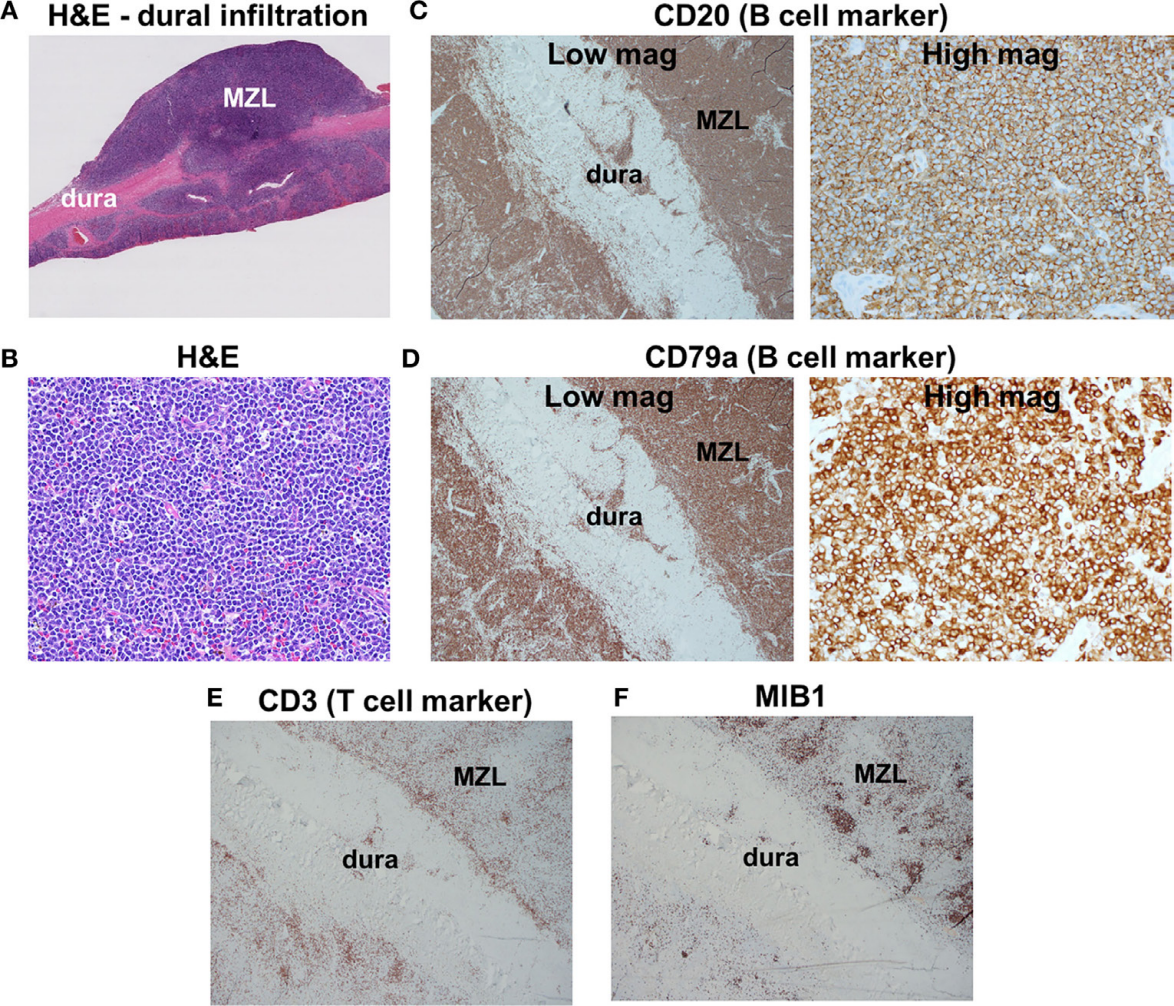
Histologic findings. **(A)** Low-magnification H&E staining reveals infiltration of dura by marginal zone lymphoma (MZL) tumor cells. **(B)** High-magnification H&E photomicrograph of the neoplasm indicates densely packed small cells. **(C,D)** Tumor cells are positive for the B cell markers CD20 and CD79a. **(E)** Scattered reactive CD3+ T cells are found within the neoplasm. **(F)** MIB1 is focally elevated in germinal centers within the neoplasm.

Pathologic examination of the tumors showed dural infiltration by small-sized lymphocytes (Figures 2A,B). Vague germinal centers were seen, which were colonized by the neoplastic lymphocytes. On flow cytometry and immunohistochemical stains, the neoplastic cells were positive for the B cell markers CD19, CD20, and CD79a (Figures 2C,D) and negative for CD5, CD10 (a marker of germinal center B cells). Tumor cells were κ light chain-restricted. CD3 immunostaining (a marker of T cells) showed few reactive T cells within the tumors (Figure 2E). CD21 highlighted few residual follicular dendritic meshworks. Staining for Epstein–Barr virus (EBV) was negative. MIB1 (Ki-67) immunolabeling was overall low (20%), but high in residual germinal centers (Figure 2F). The findings were diagnostic for extranodal MZL.

Bone marrow biopsy and aspirate showed a scant low-grade B-cell lymphoma that coexpressed the B cell markers CD19 and CD20, as well as the CLL markers CD5 and CD23, but lacked CD10 or CD38. This immunophenotype was consistent with CLL/SLL. The cells were dimly κ light chain-restricted, and the population represented about 5% of overall bone marrow cellularity. Flow cytometry of peripheral blood confirmed the diagnosis. Molecular analysis comparing the dural MZL to the CLL/SLL component in bone marrow and peripheral blood was requested. Ig gene rearrangement of the dural tumor detected a κ light chain clone of the same size (Jk) as that seen in the peripheral blood. Although these lymphomas were phenotypically different and also presented at different sites, the analysis of Ig gene rearrangement raised the possibility that the two of them shared the same clonal origin.

Starting at approximately 2 months after surgery, the patient received local proton beam radiotherapy (RT) to the right frontal and temporal fields, as well as four cycles of prophylactic intrathecal cytarabine. Multiple lumbar punctures over a 6-month period following surgery showed no evidence of tumor cells. However, 5 months after surgery, he presented with swollen right supraclavicular lymph nodes. CT of the chest and abdomen showed lymph node involvement in the right supraclavicular area and left axilla. Flow cytometry immunophenotyping of supraclavicular lymph node aspirate demonstrated two abnormal B cell populations. The larger one comprised 58% of all cells, consisted of small to medium-sized cells. It was CD45+ (pan-leukocyte marker), CD19+ (B cell marker), CD20+ (B cell marker), CD5−, CD10− (a marker of germinal center B cells), and surface kappa light chain strongly+, consistent with MZL lymph node. A second smaller cell population represented 6.3% of all cells and consisted of small cells with the following immunophenotype: CD45+, CD19+, CD20+, CD5+, CD10−, CD23+, CD38−, and surface kappa weakly+. This phenotype indicated the CLL presentation in the lymph node (also known as SLL). These two populations were compatible with the previously diagnosed dural MZL and CLL/SLL, respectively. Moreover, the presence of both MZL and SLL cells in the lymph node confirmed the diagnosis of composite lymphoma. Because both neoplastic processes are considered low-grade, the patient did not receive any additional treatment. On his most recent follow-up, 10 months after surgery, he had no new symptoms, fevers, night sweats, or additional lymph node involvement.

### Epidemiological Data

Analysis of prior literature revealed that dural MZL exhibits a marked female predilection with an approximate female:male ratio of 3:1 (76% of the cases were females). The reason behind this female predominance is unknown. The median age at presentation was 51 years (range: 28–78) (11, 12), with the majority being middle-aged females. In all but one case, MZL involved the cranial dura, while in one case it was localized to the thoracic dura (13). Patients presented with a group of symptoms that include: persistent headaches (45%), seizures (30%), visual loss (25%), dizziness (10%), ataxic gait (10%), speech deficits (8%), and hearing deficits (5%). Most of the cases were immunocompetent, and tests for EBV and human immunodeficiency virus were negative when performed (14). Four cases (4%) had an underlying autoimmune or infectious disease, including hepatitis C with immunosuppression after liver transplantation (15), Sjogren’s syndrome (16), Graves’ disease (17), and multiple sclerosis (14). In two cases, the dural MZL arose after other neoplasms [meningioma (18) and inflammatory breast cancer (19)]. In our case, CLL/SLL was diagnosed concurrently in the bone marrow and peripheral blood. We consider this to be the first reported case of composite lymphoma of dural MZL and CLL.

## Discussion

### Histopathology

Marginal zone lymphoma is a low-grade, indolent type of NHL. It arises from marginal zone B cells of secondary lymphoid follicles. It shows a spectrum of cellular constituents, including small lymphocytes, plasmacytoid cells, and plasma cells. Tumor cells show expression of pan-B cell markers (CD19, CD20, and CD79a) and lack the expression of CD5, CD10 (a marker of germinal center B cells). There are frequently a limited number of small normal CD3+ reactive T lymphocytes interspersed among the larger tumor cells. Cases with plasmacytic differentiation show restricted Ig light chain with a preponderance of κ light chain restriction (Table 1). MIB1 (Ki-67) staining is usually low, ranging from 10 to 30%, which correlates with the indolent nature of this lymphoma. All these markers are essential to distinguish MZL from other types of lymphomas, such as CLL/SLL (CD5+ and CD23+), mantle cell lymphoma (CyclinD1+, CD5+, CD23−), and follicular lymphoma (CD10+ and Bcl6+). Immunophenotypically, MZL is closely related to Waldenstrom macroglobulinemia (WM), another indolent NHL. Both entities express CD19 and CD20; however, WM mainly arises in the bone marrow and has a higher CD25 expression. Moreover, MYD88 gene mutation is detected in 90% of WM patients, while being rarely found in MZL (20).

**Table 1:** Summary of cases with central nervous system dural marginal zone lymphoma in chronological order.

**No.**	**Reference**	**Location**	**Symptoms**	**Follow-up (months)**	**Status**
1	Kumar et al. (21)	Cavernous sinus	Visual defects and numbness	63	NED
2	Kumar et al. (21)	Biparietal	Seizures	22	NED
3	Kumar et al. (21)	Frontal	Seizures and numbness	7	NED
4	Kumar et al. (21)	Tentorial	Headache, visual defects, and numbness	9	NED
5	Kumar et al. (21)	Falx	Seizures	14	NED
6	Kambham et al. (22)	Tentorium	Hearing loss and weakness	48	AWD
7	Kambham et al. (22)	Frontoparietal	Headache, visual defects, and numbness	6	AWD
8	Altundag et al. (23)	Parietal	Seizures	12	NED
9	Itoh et al. (16)	Cerebellopontine	Headache and tinnitus	24	NED
10	Sanjeevi et al. (17)	Cavernous sinus	Headache and visual defects	15	NED
11	Goetz et al. (24)	Frontoparietal	Hemiparesis	3	NED
12	Lehman et al. (25)	Falx	Seizures and speech defects	8	AWD
13	Vazquez et al. (26)	Frontotemporal	Seizures	NA	NA
14	Bodi et al. (27)	Frontal	Seizures and dizziness	18	NED
15	Benouaich et al. (28)	Frontoparietal	Headache	24	NED
16	Benouaich et al. (28)	Temporal and parietooccipital	Headache	12	NED
17	Lima et al. (29)	Falx and tentorium	Headache and seizures	NA	NA
18	Garcia-Serra et al. (30)	Temporal and cavernous sinus	Visual defects	78	NED
19	Rottnek et al. (31)	Occipital	Visual defects and seizures	8	NED
20	Kelley et al. (32)	Choroid plexus of lateral ventricle	Headache and seizures	12	NED
21	Tu et al. (14)	Falx	NA	NA	NA
22	Tu et al. (14)	Frontal	Seizures	90	NED
23	Tu et al. (14)	Frontal	Seizures	13	NED
24	Tu et al. (14)	Posterior fossa	NA	NA	NA
25	Tu et al. (14)	Middle fossa	NA	NA	NA
26	Tu et al. (14)	NA	NA	NA	NA
27	Tu et al. (14)	Subdural	NA	36	NED
28	Tu et al. (14)	Frontotemporal	Headache and dizziness	21	NED
29	Tu et al. (14)	Occipital	Ataxia	25	NED
30	Tu et al. (14)	Parietal	Dysarthria, facial drop, and numbness	NA	NA
31	Tu et al. (14)	Frontoparietal	Right arm pain	65	NED
32	Tu et al. (14)	Tentorium	Visual defects	45	NED
33	Tu et al. (14)	Falx	Visual defects and gait disturbance	32	NED
34	Tu et al. (14)	Sella and suprasellar cistern	Headache and visual defects	11	NED
35	Tu et al. (14)	Falx and tentorium	Headache and ear pain	20	NED
36	George et al. (33)	Frontal	Behavioral disorder, memory loss, and aphasia	36	NED
37	Abboud et al. (34)	Cerebellopontine	Hearing loss and ataxia	60	NED
38	Assaf et al. (35)	NA	NA	336	RD/death
39	Iwamoto et al. (36)	Temporoparietal	Headache and facial weakness	78	NED
40	Iwamoto et al. (36)	Frontotemporal	Seizures and visual defects	84	NED
41	Iwamoto et al. (36)	Tentorium and frontoparietal	Headache and dizziness	53	NED
42	Iwamoto et al. (36)	Tentorium	Seizures	27	NED
43	Iwamoto et al. (36)	Frontal and sphenoid sinus	Visual loss and paresthesias	6	NED
44	Iwamoto et al. (36)	Parietal	Seizures	7	NED
45	Iwamoto et al. (36)	Frontal	Headache	8	NED
46	Iwamoto et al. (36)	Frontal	Headaches	5	NED
47	Pavlou et al. (37)	Frontoparietal	Arm weakness, partial seizures, and dysphasia	NA	AWD
48	Saggioro et al. (38)	Frontotemporoparietal and tentorial	Headaches, facial weakness, and seizures	12, 24	RD/death
49	Jung et al. (39)	Choroid plexus	Seizures and paresis	NA	NA
50	Ancheta et al. (19)	Temporal and parietooccipital	Aphasia, altered mental status, and seizures	4	NED
51	Bhagavathi et al. (40)	Temporal	Speech defect and numbness	1	AWD
52	Puri et al. (41)	Tentorium	Visual defects and seizures	48	NED
53	Puri et al. (41)	Frontal	Headaches	30	NED
54	Puri et al. (41)	Convexity	Seizures	32	NED
55	Puri et al. (41)	Frontal	Headache, seizures, and visual loss	36	NED
56	Puri et al. (41)	Frontal and sphenoid sinus	Visual loss and paresthesia	8	NED
57	Razaq et al. (12)	Cavernous sinus and optic nerve	Headaches and third cranial nerve palsy	25	NED
58	Razaq et al. (12)	Posterior fossa	Headache and dizziness	30	NED
59	Razaq et al. (12)	Anterior falx	Headache and ataxic gait	23	NED
60	Razaq et al. (12)	Corpus callosum	Headaches and weakness	48	NED
61	Razaq et al. (12)	Temporoparietal	Headaches	2	Died*
62	Ferguson et al. (42)	Cavernous sinus and optic foramen	Vision loss and exophthalmos	36	NED
63	Gocmen et al. (43)	Frontotemporoparietal	Seizures and dysphasia	6	NED
64	Shaia et al. (44)	Posterior fossa	Dizziness and vomiting	6	NED
65	Matmati et al. (7)	Frontal	Visual defects	33	AWD
66	Reis et al. (45)	Cavernous sinus	Visual defects	NA	NA
67	Kamoshima et al. (46)	Frontal	Seizures	36	NED
68	Beltran et al. (47)	Parietal and occipital	Headaches	21	AWD
69	Beltran et al. (47)	Frontal	Headaches and seizures	36	NED
70	Beltran et al. (47)	Falx and superior sagittal sinus	Headache, diplopia, and vertigo	12	NED
71	Dey et al. (13)	Spinal (thoracic)	Sensory and motor	12	NED
72	Martin et al. (18)	Parietooccipital	Headache and visual defects	12	NED
73	Sebastian et al. (48)	Falx	Seizures	9	AWD
74	Sebastian et al. (48)	Choroid plexus	Headache and dizziness	12	AWD
75	Choi et al. (9)	Falx and superior sagittal sinus	Headache	33	NED
76	Neidert et al. (49)	Frontoparietal	Facial numbness and seizures	24	NED
77	Okimoto et al. (50)	Frontal and superior sagittal sinus	Headache	2	NA
78	Chen et al. (51)	Posterior fossa	Headache and blurred vision	12	AWD
79	Kihara et al. (52)	Jugular tubercle	Double vision and hemiparesis	9 NA	RD/NED
80	De la Fuente (53)	Tentorium	Seizures	145	NED
81	De la Fuente (53)	Frontoparietal	Seizures and gait disturbances	13	NED
82	De la Fuente (53)	Parietooccipital	Headache and visual defects	86	NED
83	De la Fuente (53)	Frontal	Headache	135	NED
84	De la Fuente (53)	Tentorium	Facial numbness and tinnitus	80	NED
85	De la Fuente (53)	Temporoparietal	Walking difficulty	102	NED
86	De la Fuente (53)	Occipital	Seizures and visual defects	67	NED
87	De la Fuente (53)	Temporal–frontal	Headache	NA	NA
88	De la Fuente (53)	Frontoparietal	Seizures	52	NED
89	De la Fuente (53)	Falx	Seizures	NA	NA
90	De la Fuente (53)	Frontal and parietal	Seizures	9	NED
91	De la Fuente (53)	Temporoparietal	Headache and facial weakness	209	NED
92	De la Fuente (53)	Frontal and orbital	Visual defects	9	RD/NED
93	De la Fuente (53)	Temporal–frontal	Headache, seizures, and visual defects	37	RD/NED
94	De la Fuente (53)	Frontal	Headache	15	NED
95	De la Fuente (53)	Cavernous sinus	Facial pain	20	RD/NED
96	De la Fuente (53)	Frontal	Seizures	66	NED
97	De la Fuente (53)	Temporal	Seizures	63	NED
98	De la Fuente (53)	Frontal	Seizures	56	NED
99	De la Fuente (53)	Cavernous sinus	Facial numbness	36	RD/NED
100	De la Fuente (53)	Temporal	Headache and seizures	29	NED
101	De la Fuente (53)	Suprasellar	Headache	21	NED
102	De la Fuente (53)	Cavernous sinus	Cranial nerve palsy	10	NED
103	De la Fuente (53)	Cerebellopontine	Gait disturbances	8	NED
104	De la Fuente (53)	Cavernous sinus	Cranial nerve palsy	2	NED
105	Present case	Frontotemporal	Headaches, imbalance, and pronator drift	10	AWD

*NED, no evidence of disease; AWD, alive with the disease; RD, relapsed disease; NA, not available*.

*Death was attributed to a cause other than lymphoma.

The pathogenesis of dural MZL is not well delineated. In the CNS, there is no MALT tissue. However, it has been hypothesized that meningothelial cells are analogous to epithelial cells at sites where extranodal MZL arises (16, 21, 40). Meningothelial cells are found throughout the arachnoid membrane and are concentrated in the arachnoid villi adjacent to the dural venous sinuses. Indeed, convexity dura is the most common site of dural MZL (12).

### Cytogenetic and Molecular Studies

Extranodal MZL, especially in the gastric, intestinal, and pulmonary tissues, has a characteristic t(11;18)(q21;q21) translocation (54, 55), where the 3′ end of the *MALT1* gene on chromosome 18 is fused to the 5′ portion of *API2*, located on chromosome 11. This translocation is considered the most common chromosomal aberration in extranodal MZL (55); however, it is not found in the nodal and splenic types (14, 54, 55). Another common abnormality is the t(14;18)(q32;q21) translocation, which occurs in 15–20% of extranodal MZL, especially in non-gastrointestinal sites (54). It brings the *MALT1* gene under the control of the Ig heavy chain enhancer on chromosome 14 (55, 56). These two translocations lead to upregulation of BCL10, a protein component of a signaling complex that activates NF-κB and promotes the growth and survival of B cells. Trisomy of chromosomes 3, 7, 12, and 18 can be found in all types of MZL (14, 57), but their effect on lymphomagenesis is still unclear (58). In our review of dural MZL, we observed that trisomy 3 is the most common chromosomal aberration (14, 59), followed by t(14;18)(q32;q21) (40). Interestingly, none of the reported cases had t(11;18).

### Imaging Studies

Dural-based MZL is often misdiagnosed as meningioma on imaging studies, because, both tumors appear as enhancing extra-axial lesions. By contrast, the diffuse large B-cell lymphoma (DLBCL) variant of primary CNS high-grade lymphoma typically localizes to the brain parenchyma. Dural MZL frequently shows a “dural tail” sign on gadolinium-enhanced MRI, a finding classically seen with meningioma (48). It can also induce vasogenic edema, mass effect, and ventricular compression. Although the anatomic location of dural involvement is variable, MZL most commonly arises at the hemispheric convexities, interhemispheric falx, tentorium, and cavernous sinus.

### Treatment and Prognosis

The optimal management of non-gastric extranodal MZL, and particularly dural MZL is not clearly defined. Surgery, chemotherapy, and RT alone or in combination have been used (60). MZL is indolent in nature and thus has a favorable prognosis. Treatment paradigms for dural MZL have varied (Table 2), probably due to the paucity of cases compared with the gastric and the other extranodal sites.

**Table 2:** Phenotypic and cytogenetic markers in cases with dural marginal zone lymphoma.

**Feature**	**Positive**	**Negative**
CD19/CD20	105/105 (100%)	–
CD79a	21/21 (100%)	–
CD5	–	59/59 (100%)
CD3	3/29 (10%)	26/29 (90%)
CD10	–	22/22 (100%)
CD23	5/31 (16%)	26/31 (84%)
Bcl2	19/21 (90%)	2/21 (10%)
CyclinD1	–	33/33 (100%)
CD43	9/13 (69%)	4/13 (31%)
IgL κ	32/42 (76%)	–
IgL λ	10/42 (24%)	–
IgG4	7/21 (33%)	14/21 (67%)
Trisomy 3	8/17 (47%)	–
IgH-MALT1	1/17 (6%)	–

All patients in our review underwent some form of surgical intervention, either biopsy to obtain tissue and establish diagnosis or resection of the tumor. The resection was complete in many cases; however, partial resection was done in some cases due to neuroanatomic constraints. RT has been shown to significantly prolong overall survival (OS) in both gastric and non-gastric extranodal MZL and was used in 70% of the dural MZL patients (61). Chemotherapy was also utilized in the management of 35% of the cases, where methotrexate was the most commonly used agent, either alone or as part of combinatorial therapy (41). Rituximab, a monoclonal antibody against CD20, has also been frequently used in treating dural MZL. It has shown significant activity and tolerability in both gastric and non-gastric extranodal MZL (62). Most of the patients (64%) were treated with two modalities including resection and RT (72%); resection and chemotherapy (17%) or RT plus chemotherapy (11%). On the other hand, 27% were treated with a single modality like resection (44%), RT (37%), or chemotherapy (19%) alone. Finally, only 9% of patients are treated with all three modalities combined.

Dural MZL has an excellent prognosis. In the 105 patients we reviewed, the median follow-up time was 23 months (range 1–336 months). There was disease recurrence in 12 cases: 4 were at the same site (13, 14, 38, 52), while another 4, including this case, were extracranial and with no evidence of CNS involvement (7, 35, 41). The information of the other four relapsed cases was unavailable. The median and average time to relapse was 12 and 40 months, respectively. However, the majority of patients achieved complete remission on further follow-up. Three patients expired, either due to toxicity from the treatment or due to causes other than MZL. One of the cases was diagnosed with a recurrence and died from pneumonia and sepsis secondary to adjuvant therapy (38), while the other two cases succumbed after 2 and 6 months of treatment, respectively. Their death was attributed to causes other than lymphoma (12, 35).

**Figure 3:**
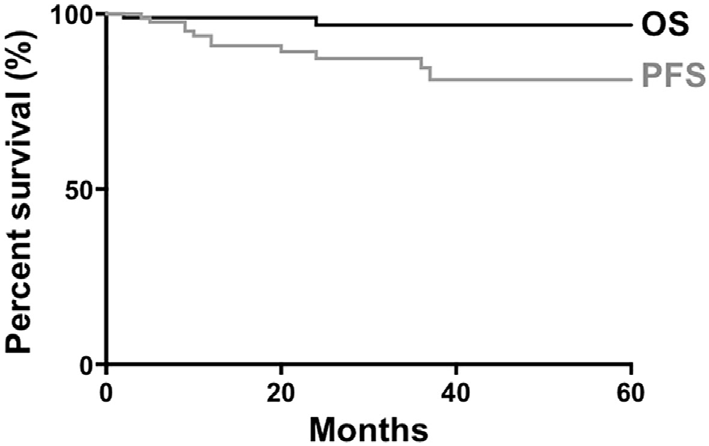
Survival of patients with dural marginal zone lymphoma. Data from 93 patients were available for generation of curves for overall survival (OS) and progression-free survival (PFS).

A study on non-gastric MZL, which did not include the dural variant, reported that the 5-year OS and progression-free survival (PFS) were 90 and 60%, respectively (60). Our analysis of previous published dural MZL cases (*n* = 93) showed that the 5-year OS and PFS were 96.7 and 81.2%, respectively (Figure 3), suggesting a better prognosis compared with extranodal MZL in other tissues.

### Comparison With Other Lymphomas

High-grade DLBCL of brain parenchyma is the most common type of primary CNS lymphomas, followed by Burkitt lymphoma (63). Low-grade CNS lymphomas are much less common and are associated with a better prognosis compared with DLBCL. Dural MZL is the most common type of low-grade CNS lymphoma. It occurs in immune-competent patients, and middle-aged females are predominantly affected, in contrast to the immunocompromised state and male predilection seen in CNS DLBCL (14). Most of the large studies on non-gastric MZL (55, 58, 60) did not include dural-based tumors, likely due to the rarity of such cases. Interestingly, dural MZL was not associated with an infectious etiology like the stomach, intestine, ocular adnexa, and skin extranodal MZL (58, 64). Moreover, only 5% of the dural cases were associated with chronic inflammatory or autoimmune conditions.

### Composite Lymphoma

The case we encountered is unique in its presentation as a composite lymphoma. When two distinct types of lymphomas occur in the same patient, the disease state is termed composite lymphoma. This term was introduced in 1954 by Muller-Hermelink et al. (65) and then refined by Kim et al. (66). Composite lymphomas can be composed of a Hodgkin’s lymphoma and an NHL, or two distinct NHL tumors. It may occur in one lymph node or multiple sites in one patient. There can be sharp or diffuse borders or even partial mixtures of infiltrates of various lymphoma types if they occur at the same site. Most of the reported cases of composite NHLs were mantle cell or follicular cell lymphomas associated with CLL or DLBCL (10, 66, 67). In most reported cases, the two lymphomas were not clonally related. However, in a few cases, Ig gene rearrangement analysis suggested a common clonal origin of two morphologically distinct types (10, 67), as occurred in this case. With advances in next-generation sequencing and molecular pathology, the question of a common origin in composite lymphoma can be definitively addressed in the future.

The case we present here is the first report of composite lymphoma consisting of dural MZL and CLL/SLL. The two components initially presented at different sites, but then concurrently invaded systemic lymph nodes.

## Conclusion

Primary dural extranodal MZL is a low-grade NHL. Appropriate pathology and immunophenotyping by IHC and flow cytometry are essential for establishing the diagnosis and distinguishing this neoplasm from other lymphomas or primary brain tumors. Combinations of surgical resection, RT, and chemotherapy are effective in producing complete remission in most cases. Cases of composite lymphoma, where the dural MZL spreads concurrently with another lymphoma, require monitoring of both neoplastic processes.

## Author Contributions

MB and DP conceived the study, collected data, and wrote the manuscript. BL, DZ, and CL provided pathology data and helped write the manuscript. TS, DG, and BR provided clinical data and helped write the manuscript.

## Conflict of Interest Statement

The authors declare that the research was conducted in the absence of any commercial or financial relationships that could be construed as a potential conflict of interest.

## References

[1] Herman TS Hammond N Jones SE Butler JJ Byrne GE Jr Mckelvey EM Involvement of the central nervous system by non-Hodgkin’s lymphoma: the Southwest Oncology Group experience. Cancer (1979) 43:390–7. 10.1002/1097-0142(197901)43:1<390::AID-CNCR2820430155>3.0.CO;2-U 761173

[2] Ferreri AJ Marturano E. Primary CNS lymphoma. Best Pract Res Clin Haematol (2012) 25:119–30. 10.1016/j.beha.2011.12.001 22409828

[3] Ostrom QT Gittleman H Fulop J Liu M Blanda R Kromer C CBTRUS statistical report: primary brain and central nervous system tumors diagnosed in the United States in 2008-2012. Neuro Oncol (2015) 17(Suppl 4):iv1–62. 10.1093/neuonc/nov189 26511214 PMC4623240

[4] Jakic-Razumovic J Aurer I. The World Health Organization classification of lymphomas. Croat Med J (2002) 43:527–34. 12402390

[5] Vannata B Stathis A Zucca E Management of the marginal zone lymphomas. In: Evens MA Blum AK, editors. Non-Hodgkin Lymphoma: Pathology, Imaging, and Current Therapy. Cham: Springer International Publishing (2015). p. 227–49. 10.1007/978-3-319-13150-4_9 25655612

[6] Isaacson P Wright DH. Malignant lymphoma of mucosa-associated lymphoid tissue. A distinctive type of B-cell lymphoma. Cancer (1983) 52:1410–6. 10.1002/1097-0142(19831015)52:8<1410::AID-CNCR2820520813>3.0.CO;2-3 6193858

[7] Matmati K Matmati N Hannun YA Rumboldt Z Patel S Lazarchick J Dural MALT lymphoma with disseminated disease. Hematol Rep (2010) 2:e10. 10.4081/hr.2010.e10 22184513 PMC3222263

[8] Dreyling M Thieblemont C Gallamini A Arcaini L Campo E Hermine O ESMO Consensus conferences: guidelines on malignant lymphoma. Part 2: marginal zone lymphoma, mantle cell lymphoma, peripheral T-cell lymphoma. Ann Oncol (2013) 24:857–77. 10.1093/annonc/mds643 23425945

[9] Choi JY Chung JH Park YJ Jung GY Yoon TW Kim YJ Extranodal marginal zone b-cell lymphoma of mucosa-associated tissue type involving the dura. Cancer Res Treat (2015) 48(2):859–63. 10.4143/crt.2014.334 26194368 PMC4843722

[10] Küppers R Dührsen U Hansmann M-L. Pathogenesis, diagnosis, and treatment of composite lymphomas. Lancet Oncol (2014) 15:e435–46. 10.1016/S1470-2045(14)70153-6 25186047

[11] Park I Huh J Kim JH Lee SW Ryu MH Kang YK. Primary central nervous system marginal zone B-cell lymphoma of the basal ganglia mimicking low-grade glioma: a case report and review of the literature. Clin Lymphoma Myeloma (2008) 8:305–8. 10.3816/CLM.2008.n.043 18854286

[12] Razaq W Goel A Amin A Grossbard ML. Primary central nervous system mucosa-associated lymphoid tissue lymphoma: case report and literature review. Clin Lymphoma Myeloma (2009) 9:E5–9. 10.3816/CLM.2009.n.052 19525185

[13] Dey M Daniel S Wong RH Smith SM Yamini B. Marginal zone lymphoma of the thoracic dura causing spinal cord compression. J Clin Neurosci (2013) 20:171–3. 10.1016/j.jocn.2012.01.042 22989787

[14] Tu PH Giannini C Judkins AR Schwalb JM Burack R O’neill BP Clinicopathologic and genetic profile of intracranial marginal zone lymphoma: a primary low-grade CNS lymphoma that mimics meningioma. J Clin Oncol (2005) 23:5718–27. 10.1200/JCO.2005.17.624 16009945

[15] Estevez M Chu C Pless M. Small B-cell lymphoma presenting as diffuse dural thickening with cranial neuropathies. J Neurooncol (2002) 59:243–7. 10.1023/A:1019913611512 12241122

[16] Itoh T Shimizu M Kitami K Kamata K Mitsumori K Fujita M Primary extranodal marginal zone B-cell lymphoma of the mucosa-associated lymphoid tissue type in the CNS. Neuropathology (2001) 21:174–80. 10.1046/j.1440-1789.2001.00392.x 11666014

[17] Sanjeevi A Krishnan J Bailey PR Catlett J. Extranodal marginal zone B-cell lymphoma of malt type involving the cavernous sinus. Leuk Lymphoma (2001) 42:1133–7. 10.3109/10428190109097736 11697633

[18] Martin SE Khalidi HS Hattab EM. Marginal zone B-cell lymphoma involving a longstanding fibrous meningioma: an initial manifestation of systemic disease. Hum Pathol (2013) 44:2609–13. 10.1016/j.humpath.2013.04.016 23850496

[19] Ancheta RG Lewin H Said J Hurvitz SA Primary dural marginal zone lymphoma in a woman with inflammatory breast cancer. J Clin Oncol (2008) 26:326–8. 10.1200/JCO.2007.14.0236 18182673

[20] Ghobrial IM. Are you sure this is Waldenstrom macroglobulinemia? Hematology Am Soc Hematol Educ Program (2012) 2012:586–94. 10.1182/asheducation-2012.1.586 23233639

[21] Kumar S Kumar D Kaldjian EP Bauserman S Raffeld M Jaffe ES. Primary low-grade B-cell lymphoma of the dura: a mucosa associated lymphoid tissue-type lymphoma. Am J Surg Pathol (1997) 21:81–7. 10.1097/00000478-199701000-00009 8990144

[22] Kambham N Chang Y Matsushima AY. Primary low-grade B-cell lymphoma of mucosa-associated lymphoid tissue (MALT) arising in dura. Clin Neuropathol (1998) 17:311–7. 9832258

[23] Altundag MK Ozisik Y Yalcin S Akyol F Uner A. Primary low grade B-cell lymphoma of the dura in an immunocompetent patient. J Exp Clin Cancer Res (2000) 19:249–51. 10965827

[24] Goetz P Lafuente J Revesz T Galloway M Dogan A Kitchen N. Primary low-grade B-cell lymphoma of mucosa-associated lymphoid tissue of the dura mimicking the presentation of an acute subdural hematoma. Case report and review of the literature. J Neurosurg (2002) 96:611–4. 10.3171/jns.2002.96.3.0611 11883850

[25] Lehman NL Horoupian DS Warnke RA Sundram UN Peterson K Harsh GRT. Dural marginal zone lymphoma with massive amyloid deposition: rare low-grade primary central nervous system B-cell lymphoma. Case report. J Neurosurg (2002) 96:368–72. 10.3171/jns.2002.96.2.0368 11838814

[26] Vazquez A Portillo E Guridi J Zazpe I Caballero MC Valenti C. [Primary low-grade non-Hodgkin’s B lymphoma mimicking meningioma]. Neurocirugia (Astur) (2002) 13:50–3. 10.1016/S1130-1473(02)70653-2 11939095

[27] Bodi I Hussain A Gullan RW Safa AS. January 2003: 56-year-old female with right frontal tumor of the dura. Brain Pathol (2003) 13(417–418):423. 12946031

[28] Benouaich A Delord JP Danjou M Richaud J Urocoste E Soum F [Primary dural lymphoma: a report of two cases with review of the literature]. Rev Neurol (Paris) (2003) 159:652–8. RNE-07-2003-159-6-7-0000-0000-101019-ART67 12910073

[29] Lima VS Leite EB Fonseca RP Fernandes AS Jr Patients presenting with CNS lesions. Case 1. Primary low-grade mucosa-associated B-cell lymphoma of the dura. J Clin Oncol (2003) 21:4058–60. 10.1200/JCO.2003.12.054 14581428

[30] Garcia-Serra A Price Mendenhall N Hinerman RW Lynch JW Jr Braylan RC Mancuso AA. Management of neurotropic low-grade B-cell lymphoma: report of two cases. Head Neck (2003) 25:972–6. 10.1002/hed.10311 14603459

[31] Rottnek M Strauchen J Moore F Morgello S. Primary dural mucosa-associated lymphoid tissue-type lymphoma: case report and review of the literature. J Neurooncol (2004) 68:19–23. 10.1023/B:NEON.0000024704.70250.42 15174517

[32] Kelley TW Prayson RA Barnett GH Stevens GH Cook JR Hsi ED. Extranodal marginal zone B-cell lymphoma of mucosa-associated lymphoid tissue arising in the lateral ventricle. Leuk Lymphoma (2005) 46:1423–7. 10.1080/10428190500205895 16194887

[33] George AC Ozsahin M Janzer R Agassis S Meuli R Baur AS Primary intracranial dural lymphoma of mucosa-associated lymphoid tissue (MALT) type: report of one case and review of the literature. Bull Cancer (2005) 92:E51–6. 16122999

[34] Abboud H Carpentier A Martin-Duverneuil N Kujas M Hoang-Xuan K MALT lymphoma presenting as a meningioma. J Neurooncol (2005) 75:221 10.1007/s11060-005-1940-2 16283446

[35] Assaf C Coupland SE Hummel M Jahnke K Mostafafivar S Stein H Relapse of primary extranodal marginal-zone B-cell lymphoma of the dura mater. Lancet Oncol (2005) 6:187–9. 10.1016/S1470-2045(05)01771-7 15737836

[36] Iwamoto FM Deangelis LM Abrey LE. Primary dural lymphomas: a clinicopathologic study of treatment and outcome in eight patients. Neurology (2006) 66:1763–5. 10.1212/01.wnl.0000218284.23872.eb 16769960

[37] Pavlou G Pal D Bucur S Chakrabarty A Van Hille PT. Intracranial non-Hodgkin’s MALT lymphoma mimicking a large convexity meningioma. Acta Neurochir (Wien) (2006) 148:791–3; discussion 793. 10.1007/s00701-006-0761-1 16570114

[38] Saggioro FP Colli BO Paixao-Becker AN De Rezende GG Santos AC Neder L Primary low-grade MALT lymphoma of the dura. Histopathology (2006) 49:323–6. 10.1111/j.1365-2559.2006.02433.x 16918987

[39] Jung TY Jung S Lee MC Lee KH. Extranodal marginal zone B-cell lymphoma mimicking meningioma in lateral ventricle: a case report and possible pathogenesis. J Neurooncol (2006) 80:63–7. 10.1007/s11060-006-9153-x 16628474

[40] Bhagavathi S Greiner TC Kazmi SA Fu K Sanger WG Chan WC. Extranodal marginal zone lymphoma of the dura mater with IgH/MALT1 translocation and review of literature. J Hematop (2008) 1:131–7. 10.1007/s12308-008-0005-9 19669212 PMC2713483

[41] Puri DR Tereffe W Yahalom J. Low-dose and limited-volume radiotherapy alone for primary dural marginal zone lymphoma: treatment approach and review of published data. Int J Radiat Oncol Biol Phys (2008) 71:1425–35. 10.1016/j.ijrobp.2007.12.007 18234430

[42] Ferguson SD Musleh W Gurbuxani S Shafizadeh SF Lesniak MS. Intracranial mucosa-associated lymphoid tissue (MALT) lymphoma. J Clin Neurosci (2010) 17:666–9. 10.1016/j.jocn.2009.10.001 20202849

[43] Gocmen S Gamsizkan M Onguru O Sefali M Erdogan E. Primary dural lymphoma mimicking a subdural hematoma. J Clin Neurosci (2010) 17:380–2. 10.1016/j.jocn.2009.02.014 20079653

[44] Shaia J Kerr PB Saini A Roberti F Kapil J Jones R Mucosa-associated lymphoma tissue of the dura presenting as meningioma. South Med J (2010) 103:950–2. 10.1097/SMJ.0b013e3181eb3477 20689487

[45] Reis F Schwingel R Queiroz Lde S Zanardi Vde A Primary dural lymphoma: a rare subtype of primary central nervous system lymphoma (PCNSL). Arq Neuropsiquiatr (2011) 69:264–5. 10.1590/S0004-282X2011000200025 21537574

[46] Kamoshima Y Sawamura Y Sugiyama T Yamaguchi S Houkin K Kubota K Primary central nervous system mucosa-associated lymphoid tissue lymphoma – case report. Neurol Med Chir (Tokyo) (2011) 51:527–30. 10.2176/nmc.51.527 21785250

[47] Beltran BE Kuritzky B Quinones P Morales D Alva JC Lu G Extranodal marginal zone lymphoma of the cranial dura mater: report of three cases and systematic review of the literature. Leuk Lymphoma (2013) 54:2306–9. 10.3109/10428194.2013.771399 23363270

[48] Sebastian C Vela AC Figueroa R Marin MA Alfaro J. Primary intracranial mucosa-associated lymphoid tissue lymphoma. A report of two cases and literature review. Neuroradiol J (2014) 27:425–30. 10.15274/NRJ-2014-10074 25196615 PMC4236878

[49] Neidert MC Leske H Burkhardt JK Rushing EJ Bozinov O A 44-year old male with right-sided facial numbness. Dura-associated extranodal marginal zone B cell lymphoma (MALT lymphoma). Brain Pathol (2015) 25:113–4. 10.1111/bpa.12234 25521183 PMC8029347

[50] Okimoto RA Perry A Rubenstein JL 77-year-old woman with a dural-based mass. Marginal zone B-cell lymphoma (MZBCL). Brain Pathol (2015) 25:111–2. 10.1111/bpa.12233 25521182 PMC4942276

[51] Chen J Yan Z Zeng H Li H Han A Teaching neuroimages: primary dural mucosa-associated lymphoid tissue lymphoma. Neurology (2015) 84:e107–8. 10.1212/WNL.0000000000001457 25847001

[52] Kihara K Sato M Kado K Fukuda K Nakamura T Yamakami I [A case of primary dural lymphoma of jugular tubercle mimicking lower clival meningioma]. No Shinkei Geka (2016) 44:391–6. 10.11477/mf.1436203297 27166844

[53] De La Fuente MI Haggiagi A Moul A Young RJ Sidani C Markoe A Marginal zone dural lymphoma: the Memorial Sloan Kettering Cancer Center and University of Miami experiences. Leuk Lymphoma (2017) 58:882–8. 10.1080/10428194.2016.1218006 27649904 PMC5576515

[54] Troppan K Wenzl K Neumeister P Deutsch A. Molecular pathogenesis of MALT lymphoma. Gastroenterol Res Pract (2015) 2015:102656. 10.1155/2015/102656 25922601 PMC4397421

[55] Raderer M Kiesewetter B Ferreri AJ Clinicopathologic characteristics and treatment of marginal zone lymphoma of mucosa-associated lymphoid tissue (MALT lymphoma). CA Cancer J Clin (2016) 66:152–71. 10.3322/caac.21330 26773441

[56] Streubel B Lamprecht A Dierlamm J Cerroni L Stolte M Ott G T(14;18)(q32;q21) involving IGH and MALT1 is a frequent chromosomal aberration in MALT lymphoma. Blood (2003) 101:2335–9. 10.1182/blood-2002-09-2963 12406890

[57] Maes B De Wolf-Peeters C Marginal zone cell lymphoma – an update on recent advances. Histopathology (2002) 40:117–26. 10.1046/j.1365-2559.2002.01360.x 11952855

[58] Zucca E Bertoni F. The spectrum of MALT lymphoma at different sites: biological and therapeutic relevance. Blood (2016) 127:2082–92. 10.1182/blood-2015-12-624304 26989205

[59] Venkataraman G Rizzo KA Chavez JJ Streubel B Raffeld M Jaffe ES Marginal zone lymphomas involving meningeal dura: possible link to IgG4-related diseases. Mod Pathol (2011) 24:355–66. 10.1038/modpathol.2010.206 21102421 PMC7425666

[60] Zucca E Conconi A Pedrinis E Cortelazzo S Motta T Gospodarowicz MK Nongastric marginal zone B-cell lymphoma of mucosa-associated lymphoid tissue. Blood (2003) 101:2489–95. 10.1182/blood-2002-04-1279 12456507

[61] Goda JS Gospodarowicz M Pintilie M Wells W Hodgson DC Sun A Long-term outcome in localized extranodal mucosa-associated lymphoid tissue lymphomas treated with radiotherapy. Cancer (2010) 116:3815–24. 10.1002/cncr.25226 20564130

[62] Conconi A Martinelli G Thieblemont C Ferreri AJ Devizzi L Peccatori F Clinical activity of rituximab in extranodal marginal zone B-cell lymphoma of MALT type. Blood (2003) 102:2741–5. 10.1182/blood-2002-11-3496 12842999

[63] Brastianos PK Batchelor TT. Primary central nervous system lymphoma: overview of current treatment strategies. Hematol Oncol Clin North Am (2012) 26:897–916. 10.1016/j.hoc.2012.05.003 22794289

[64] Thieblemont C De La Fouchardière A Coiffier B. Nongastric mucosa-associated lymphoid tissue lymphomas. Clin Lymphoma (2003) 3:212–24. 10.3816/CLM.2003.n.002 12672270

[65] Muller-Hermelink HK Zettl A Pfeifer W Ott G. Pathology of lymphoma progression. Histopathology (2001) 38:285–306. 10.1046/j.1365-2559.2001.01120.x 11318894

[66] Kim H Hendrickson R Dorfman RF Composite lymphoma. Cancer (1977) 40:959–76. 10.1002/1097-0142(197709)40:3<959::AID-CNCR2820400302>3.0.CO;2-3 332325

[67] Hoeller S Zhou Y Kanagal-Shamanna R Xu-Monette ZY Hoehn D Bihl M Composite mantle cell lymphoma and chronic lymphocytic leukemia/small lymphocytic lymphoma: a clinicopathologic and molecular study. Hum Pathol (2013) 44:110–21. 10.1016/j.humpath.2012.04.022 22944294 PMC3640316

